# The direct prognosis comparison of ^125^I low-dose-rate brachytherapy versus laparoscopic radical prostatectomy for patients with intermediate-risk prostate cancer

**DOI:** 10.1186/s40001-023-01140-4

**Published:** 2023-06-02

**Authors:** Zhen Liang, Chen Yuliang, Ming Zhu, Yi Zhou, Xingcheng Wu, Hanzhong Li, Bu Fan, Zhien Zhou, Weigang Yan

**Affiliations:** 1 Department of Urology, Peking Union Medical College Hospital, Peking Union Medical College, Chinese Academy of Medical Sciences, Beijing, China; 2 Department of Urology, Surgical Building of Peking, Union Medical College Hospital, No.1 Shuaifuyuan, Wangfujing, Dong Cheng District, Beijing, 100730 China

**Keywords:** Prostatic neoplasms, Low-dose-rate brachytherapy, Radical prostatectomy, Treatment outcomes, Comparative effectiveness

## Abstract

**Background:**

This study aims to compare the clinical outcomes after performing radical prostatectomy (RP) or low-dose-rate brachytherapy (LDR) for patients with intermediate-risk prostate cancer (IRPC).

**Methods:**

We performed a retrospective analysis on 361 IRPC patients who underwent treatment in Peking Union Medical College Hospital from January 2014 to August 2021, of which 160 underwent RP and 201 underwent Iodine-125 LDR. Patients were followed in clinic monthly during the first three months and at three-month intervals thereafter. Univariate and multivariate regression analyses were conducted to predict biochemical relapse-free survival (bRFS), clinical relapse-free survival (cRFS), cancer-specific survival (CSS), and overall survival (OS). Biochemical recurrence was defined using the Phoenix definition for LDR and the surgical definition for RP. The log-rank test was applied to compare bRFS between the two modalities, and Cox regression analysis was performed to identify factors associated with bRFS.

**Results:**

Median follow-up was 54 months for RP and 69 months for LDR. According to log-rank test, the differences of 5-year bRFS (70.2% vs 83.2%, P = 0.003) and 8-year bRFS (63.1% vs 68.9%, P < 0.001) between RP and LDR groups were statistically significant. Our results also indicated that there was no significant difference in terms of cRFS, CSS, or OS between the two groups. With multivariate analysis of the entire cohort, prostate volume ≤ 30 ml (*P* < 0.001), positive margin (*P* < 0.001), and percentage positive biopsy cores > 50% (*P* < 0.001) were independent factors suggestive of worse bRFS.

**Conclusions:**

LDR is a reasonable treatment option for IRPC patients, yielding improved bRFS and equivalent rates of cRFS, CSS and OS when compared with RP.

## Introduction

Prostate cancer (PCa) is the second most common cause of cancer-related death in men globally [1]. Among male citizens in the United States, PCa accounted for 191, 930 out of approximately 893, 660 expected new cancer cases in 2020 [2]. Moreover, age, ethnicity, geographical location, family history and genetic changes are recognized risk factors for PCa and a substantial inherited component has been observed in 40–50% of PCa with several genetic mutations, such as BRCA1 and BRCA2 [3].

Previous studies have proved that active surveillance (AS) is a safe and feasible option for low-risk and carefully selected intermediate-risk PCa (IRPC) patients with a favorable long-term prognosis, low rate of metastases and rare PCa specific death. However, there remains uncertainty regarding optimal candidates and surveillance strategies, recent guidelines by the American Society of Clinical Oncology continue to favor definitive treatment for IRPC [4]. Radical prostatectomy (RP), brachytherapy, and external beam radiation therapy (EBRT) are the three primary definitive treatment options for IRPC [5]. Established clinical guidelines advocate that decisions regarding treatment options should be based on a comprehensive evaluation of tumor features, baseline prostate-specific antigen (PSA) levels, patient age, comorbidity, life expectancy, and quality of life [6–8]. However, the suitability of each treatment for patients with localized PCa remains debatable.

The preliminary investigations have indicated that low-dose-rate brachytherapy (LDR) has exhibited remarkable outcomes over a period of 10 years [9, 10]. However, since the majority of the patients were deemed to be low-risk, there is not any notable advantage of local therapy. However, as most of these patients were considered to be low-risk, there is no significant benefit to local therapy [11]. According to National Comprehensive Cancer Network (NCCN) guidelines, IRPC patients are recommended for local therapy [8]. Nevertheless, there is no randomized trial of IRPC patients to make a direct comparison between RP and LDR. Thus, evidence to inform clinical decisions regarding treatment for IRPC patients remains to be inadequate.

In present study, we aim to perform a retrospective study of IRPC to compare the long-term outcomes of RP vs LDR in China. Moreover, this study also sought to identify variables that may predict differences in biochemical control in accordance with the most recent consensus definitions of biochemical failure (BF).

## Methods

### Patients

A total of 361 consecutive IRPC patients treated with curative intent between January 2014 and August 2021 at the Peking Union Medical College Hospital were identified, of which 160 (44.3%) underwent RP, and 201 (55.7%) received LDR. Patients were categorized according to the NCCN risk classification criteria [8], which defines IRPC by clinical stage T2b-c, Gleason score (GS) 3 + 4 (group 2) or 4 + 3 (group 3), and/or initial PSA (iPSA) of 10.1–20.0 ng/ml. Percentage positive biopsy cores (PPBC) > 50% was calculated from the pathology report. Favorable IRPC was described as patients with no more than one intermediate adverse risk factor, such as GS 3 + 4 (group 2), iPSA 10.1–20.0 ng/ml, or clinical stage T2b-c, PPBC ≤ 50%. On the other hand, those with multiple intermediate adverse risk factors, which included PPBC > 50%, or any IRPC with GS 4 + 3 (group 3), were classified as unfavorable IRPC [12]. Institutional Review Board has approved our protocols.

The following information of all patients was evaluated: medical history, physical examination, digital rectal examination, prostate volume (PV), iPSA. Clinical stages for both RP and LDR groups are determined using a standardized TNM classification system which is evaluated according to the combination of prostate biopsy pathology report, chest radiography, bone scintigraphy, CT-scan and magnetic resonance imaging (MRI) of the pelvis before biopsy. The staging was carried out by at least two surgeons at our institution. PV was calculated as anteroposterior diameter × vertical diameter × transverse diameter × 0.52 based on MRI [13].

### Treatments

The treatment option, LDR versus RP, was decided collaboratively by both doctors and patients after discussions. The clinics will initially introduce the advantages and disadvantages, prognosis and possible complications of each treatment plan to the patient in detail. After the patient has preliminary understanding of each treatment plan, they can decide the treatment plan based on their own perspectives. Patients with relative contraindications for LDR (e.g., LUTS with International Prostate Symptom Score > 12–20, transurethral resection of the prostate defects, large median lobes, and/or gland size > 50-60 ml) were recommended to undergo RP. Patients who could not tolerate RP would be directed towards LDR. Written informed consent was obtained from all participants included in the present study. According to NCCN guidelines, patients with unfavorable risk IRPC would receive adjuvant EBRT plus ADT after LDR; whereas, patients with favorable risk IRPC would undergo LDR monotherapy. In RP group, considering the low amount of lymph node metastasis found in the favorable IRPC group, the extent of pelvic lymph node dissection (PLND) was not regularly performed based on NCCN guidelines. In unfavorable IRPC group, an extended PLND would be performed when the estimated risk for pN + exceeds 5% [14]. If a patient was diagnosed with positive margins based on whole mount pathology report obtained through RP, an adjuvant EBRT plus ADT was then conducted. EBRT was delivered through 3D-conformal radiation therapy, intensity-modulated radiation therapy, or volumetric-modulated arc therapy to the primary prostate field [15]. The planning target volume for EBRT was generated through adding an 8 mm margin surrounding the clinical target volume, except posteriorly, where the margin was limited to 3 mm. Radiotherapy dosing regimen ranged from 66 to 74 Gy. Patients with positive lymph nodes or have contraindications to radiotherapy of EBRT were treated by adjuvant ADT immediately after obtaining the RP pathology report (approximately 2 weeks after surgery) until three months after PSA nadir [16]. Patients suffered from BF would be treated by salvage ADT. The ADT type we chose is the combination of bicalutamide and goserelin.

The RP was conducted through a pure laparoscopic RP technique described by Guillonneau [17] with an extraperitoneal approach and five trocar technique by two experienced surgeons both of whom had an average of RP cases of 100 per year (WY and ZZ). The vesico-urethral anastomosis was accomplished with a running suture with Y604 (Ethicon, USA). Treatment with LDR was planned for the prostate and proximal seminal vesicles to receive 145 Gy with a 5-mm margin laterally, anteriorly, and inferiorly by two urologist with over 10 years of brachytherapy experience [18]. No margin was planned superiorly (bladder) and posteriorly (rectum). ^125^I seeds were accurately introduced into preplanned positions by a brachytherapy stepping unit MICK200 (Computerized Medical Systems, Inc, St. Louis, MO, USA) using a standard 0.5 cm brachytherapy template placed over the perineum. 1 week after implantation, dosimetric analysis was conducted by CT scan, and the D90 (defined as the minimum dose covering 90% of the prostate) was obtained for each patient.

### Follow-up and study endpoints

The day of the operation of RP/LDP was counted as the day 0. Patients were followed up monthly during the first three months and at three-month intervals thereafter. If PSA level was stable, routine follow-up was scheduled every six months from 2 years after surgery. Imaging result for each patient was generally reviewed once a year and rechecked at any time if any signs of disease progression or biochemical recurrence were observed. Biochemical relapse-free survival (bRFS) and clinical relapse-free survival (cRFS) were the primary endpoints of this study, whereas cancer-specific survival (CSS) and overall survival (OS) were the secondary endpoints. BF was defined as a PSA value ≥ 0.2 ng/mL for patients who underwent RP [19] and an increase of 2 ng/mL or > nadir PSA value (Phoenix definition) [20] for patients treated by LDR. Clinical relapse was defined as metastases identified by medical imaging, with or without localizing symptoms, or biopsy-proven local recurrence. Both distant metastasis and regional lymph node metastasis were defined as clinical recurrence in cRFS analysis. Cancer-specific mortality was defined as mortality due to PCa, noted on the death certificate alongside the biochemical and clinical information, or the presence of uncontrolled metastatic disease when the patient succumbed.

### Statistical analysis

Factors considered to influence the endpoint were recorded for baseline analysis. The mean ± SD was applied to describe data in a normal distribution, while the median and interquartile range (IQR) were applied for data in a skewed distribution. To compare the difference between groups, Chi-square test, Mann–Whitney U-test and t-test were used, respectively, for suitable variables. Log-rank tests were applied to evaluate differences between two survival curves. Cox proportional-hazard models were constructed to identify factors associated with bRFS. We performed statistical analyses through SPSS version 25.0 (SPSS Inc., Chicago, IL, USA). *P* < 0.05 was considered to be statistically significant in this study.

## Results

### Patient characteristics

Tables 1, 2 present complete pretreatment characteristics of enrolled patients. A total of 361 patients were included in the present study, comprising 201 LDR patients (55.7%) and 160 RP patients (44.3%). A total of 370 participants were initially selected retrospectively, and nine patients have been lost during follow-up: two in the RP group and seven in LDR group. The median age of the study population was 70 (IQR 65–75) years. Patients in Group RP were slightly younger 66 (IQR: 62–71) than those in Group LDR 74 (IQR: 69–77), *P* < 0.001. The median follow-up for RP and LDR was 54 and 69 months, respectively. The median duration of ADT in arms LDR and RP were 183 days (IQR 176–190) and 60 days (IQR: 58–62) (*P* < 0.001). At baseline, the median PSA level was 12.29 ng/mL (IQR 9.53–15.01). The median follow-up duration for surviving patients was 63 months (IQR 44–86). According to biopsy results, in LDR group, 42 patients (20.8%), 36 patients (17.9%), 32 patients (15.9%) and 91 patients (45.2%) were diagnosed with stages T1c, T2a, T2b, and T2c, respectively; whereas, in RP group, 15 patients (9.4%), 40 patients (25.0%), 41 patients (25.6%) and 64 patients (40.0%) were diagnosed with stages T1c, T2a, T2b, and T2c, respectively. The clinical TNM staging in RP Group is significantly higher than LDR group. In patients underwent RP, according to whole mount pathology report, positive margins were found in 55 (34.4%) patients; the extracapsular extension was found in 31 (19.3%) patients and seminal vesicle invasion in 18 (11.3%) patients, resulting in 49 (30.2%) RP patients upstaged to pathologic T3. Biochemical recurrence was observed in 46 patients in the RP group and 42 patients in the LDR group (*P* = 0.004). The median age for BF was 68 years (IQR 62–75), median PSA was 11.82 ng/ml (IQR 9.02–15.01) and the median PV was 20.96 ml (IQR 18.95–33.02). As for the clinical recurrence aspect, nine patients were found to have metastasis for both the RP group and the LDR group (*P* = 0.628). The median age of metastasis onset was 69 years (61–75), with a median PSA of 10.01 ng/ml (7.03–17.38) and a median PV of 19.72 ml (18.15–25.35); the mean D90 for the LDR group was 144 Gy (1 standard deviation = 20.58 Gy). 31.2% of patients in RP group received ADT while 94.5% in LDR, (P < 0.001). Adjuvant EBRT was used for 15 patients (9.4%) in the RP group and 5 (2.5%) in the LDR group, respectively (*P* = 0.004).

**Table 1:** Patient cohort characteristics for RP group and LDR group

Parameters	RP(*n* = 160)	LDR(*n* = 201)	*P* value
Age(years)			< 0.001
Median	66	74	
Range	48–78	50–84	
Clinical stage	*N*, %		0.002
T1c	15 (9.4%)	42 (20.8%)	
T2a	40 (25.0%)	36 (17.9%)	
T2b	41 (25.6%)	32 (15.9%)	
T2c	64 (40.0%)	91 (45.2%)	
PPBC			0.734
≤ 50%	125	154	
> 50%	35	47	
Gleason Score	*N*, %		0.086
6(3 + 3)	75 (46.8%)	79 (39.3%)	
7(3 + 4)	54 (33.7%)	63 (31.3%)	
7(4 + 3)	31 (19.3%)	59 (29.3%)	
Prostate volume(ml)			0.172
≤ 30	76	110	
> 30	84	91	
Initial PSA (ng/ml)			0.891
Median	12.0	12.5	
Range	4.2–20.1	0.8–19.9	
Risk			0.172
Favorable	45	44	
Unfavorable	115	157	
Follow-up, months			< 0.001
Median	54	69	
Range	17–114	26–117	
Duration ADT, months			< 0.001
0	110	11	
1–6	25	94	
> 6	25	96	
Adjuvant EBRT	15	5	0.004
Risk level			0.107
Favorable risk	45	44	
Unfavorable risk	115	157

**Table 2:** Patient cohort characteristics for RP group and LDR group for patients with biochemical recurrence and patients without biochemical recurrence

Parameters	recurrence(*n* = 88)	Non-recurrence(*n* = 273)
Treatment modality		
RP	46	114
LDR	42	159
Age (years)		
Median	68	61
Range	48–81	41–74
Initial PSA (ng/ml)		
Median	11.8	12.3
Range	4.45–20.2	0.83–19..73
Treatment modality	*n*, %	
RP	46 (52.3%)	114 (41.8%)
LDR	42 (47.7%)	159 (58.2%)
Clinical stage	*n*, %	
T1c	7 (7.9%)	50 (18.3%)
T2a	15 (17.0%)	61 (22.3%)
T2b	17 (19.3%)	56 (20.5%)
T2c	49 (55.6%)	106 (38.8%)
PPBC	*n*, %	
≤ 50%	52 (59.9%)	227 (83.1%)
> 50%	36 (40.9%)	46 (16.8%)
Gleason Score	*n*, %	
6(3 + 3)	45 (51.1%)	109 (39.9%)
7(3 + 4)	27 (30.6%)	90 (32.9%)
7(4 + 3)	16 (18.1%)	74 (25.2%)
Prostate volume(ml)	*n*, %	
≤ 30	58 65.9%)	77 (28.2%)
> 30	30 (34.0%)	196 (71.8%)
Risk	*n*, %	
Favorable	18 (20.45%)	71 (26.0%)
Unfavorable	70 (79.5%)	202 (73.9%)
Metastasis status	*n*, %	
Positive	17 (19.3%)	1 (0.4%)
Negative	71 (80.6%)	272 (99.6%)

### Main outcomes

**Fig. 1:**
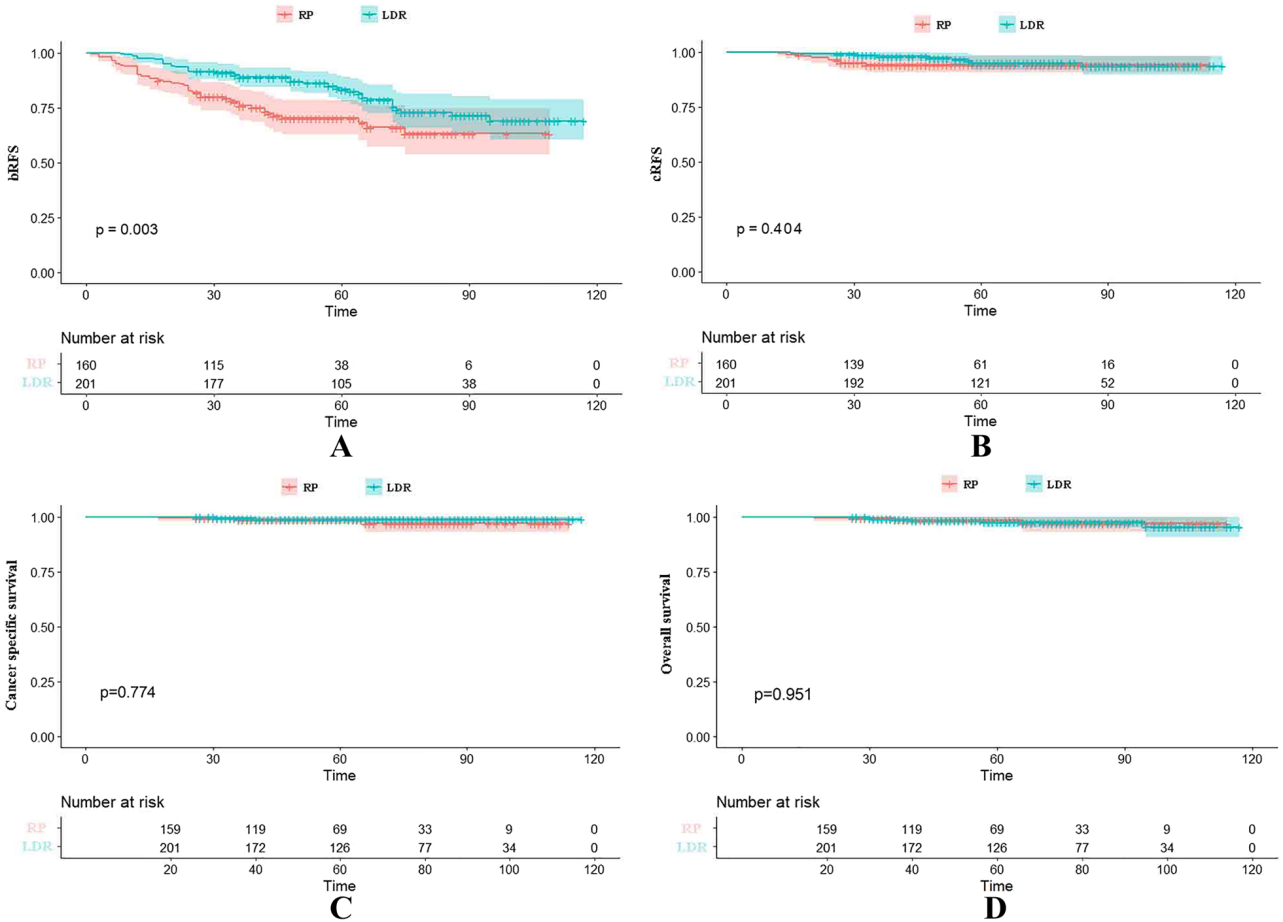
Kaplan–Meier survival curves of bRFS (**A**), cRFS (**B**), CSS (**C**) and OS (**D**) in patients with IRPC treated with LDR vs RP. *bRFS* biochemical relapse-free survival, *cRFS* clinical relapse-free survival, *CSS* cancer-specific survival, *OS* overall survival, *IRPC* intermediate-risk prostate cancer, *LDR* low-dose-rate brachytherapy, *RP* radical prostatectomy

The median time to BF was 61 months (IQR 51–81) and 44 months (IQR 27–66) for RP and LDR, respectively (*P* = 0.327). As for the median time to clinical recurrence for RP and LDR, it was 67 months (IQR 49–90) and 51 months (IQR 35–66), respectively (*P* = 0.974). The 5- and 8-year bRFS rates were 70.2% and 63.1% in the RP group and 83.2% and 68.9% in the LDR group, respectively (Fig. 1A). The log-rank test indicated that the 5- and 8-year bRFS rates for the RP group were both lower than the LDR group with *P* = 0.003 and *P* < 0.001, (Fig. 1A). The 5- and 8-year cRFS rates were 94.2% and 92.1% in the RP group and 95.1% and 93.7% in the LDR group, respectively, with *P* = 0.404 and *P* = 0.128 (Fig. 1B). The 5- and 8-year CSS rates were 99.2% and 97.4% in the RP group and 98.9% and 97% in the LDR group with *P* = 0.774 and *P* = 0.385 (Fig. 1C). The 5- and 8-year OS rates were 98.6% and 97.0% in the RP group and 97.7% and 95.4.5% in the LDR group, respectively, with *P* = 0.951 and *P* = 0.412 (Fig. 1D).

#### bRFS curves between LDR and RP

Log-rank test was used to compare the bRFS curves between LDR and RP in terms of different variables according to pretreatment characteristics. Risk of BF was significantly higher in RP patients when compared with LDR for patients with GS 7 (3 + 4: *P* = 0.027; 4 + 3: *P* = 0.001), PV > 30 ml (*P* < 0.001), iPSA ≤ 10 ng/ml (*P* = 0.007), clinical T stage with T1c–T2a (*P* = 0.016), any PPBC (≤ 50%: *P* = 0.043; > 50%: *P* = 0.003) or unfavorable IRPC (*P* = 0.003). However, survival of LDR patients was not statistically different from RP patients at any age, GS 6, PV ≤ 30 ml, iPSA > 10 ng/ml, clinical T stage with T2b–T2c or favorable IRPC.

#### Prognostic factors

Cox proportional-hazard models were constructed to identify factors associated with bRFS and CSS (Table 3). With univariate analysis of the entire cohort, treatment with RP (*P* = 0.004), age < 70 years old (*P* = 0.001), clinical T stage with T2b–T2c (*P* = 0.005), PV ≤ 30 ml (*P* < 0.001), positive margin and PPBC > 50% (*P* < 0.001) were associated with significantly worse bRFS. With multivariate analysis of the entire cohort, PV ≤ 30 ml (*P* < 0.001) and PPBC > 50% (*P* < 0.001), positive margin (*P* < 0.001) were associated with significantly worse bRFS, while other factors such as ADT application, fail to demonstrate a positive result in multivariate analysis.

**Table 3:** Univariate and multivariable analyses of prognostic factors

Factor	Univariate analysis		Multivariate analysis	
	P value	HR (95% CI)	P value	HR (95% CI)
Treatment				
RP		Ref.		Ref.
LDR	0.004	0.53 (0.35–0.81)	0.067	0.50 (0.24–1.05)
Age, years				
< 70		Ref.		Ref.
≥ 70	0.001	0.50 (0.33–0.76)	0.29	0.72 (0.40–1.31)
Clinical T stage				
T1c, T2a		Ref.		Ref.
T2b, T2c	0.005	1.99 (1.23–3.23)	0.43	1.29 (0.69–2.39)
iPSA, ng/ml				
≤ 10		Ref.	–	–
10.1–20	0.15	0.73 (0.47–1.12)	–	–
Gleason score				
6(3 + 3)		Ref.	–	–
7(3 + 4)	0.52	0.86 (0.53–1.38)	–	–
7(4 + 3)	0.095	0.62 (0.35–1.09)	–	–
Prostate volume, ml				
≤ 30		Ref.		Ref.
> 30	< 0.001	0.35 (0.22–0.56)	< 0.001	0.33 (0.18–0.60)
PPBC				
≤ 50%		Ref.		Ref.
> 50%	< 0.001	2.83 (1.85–4.33)	< 0.001	1.19 (1.71–5.95)
Duration ADT, months				
0		Ref.		
1–6	0.254	0.72 (0.65–1.29)	0.106	2.61 (0.87–4.13)
> 6	0.177	0.59 (0.51–0.71)	0.426	0.63 (0.62–3.13)
Surgical margin status				
Positive	< 0.001	2.51 (1.54–3.78)	< 0.001	2.64 (1.20–4.18)
Negative		Ref.		
Risk level				
Favorable risk	0.492	1.23 (0.68–2.21)	0.944	0.97 (0.39–2.41)
Unfavorable risk				

## Discussion

Currently, the guidelines of the American Urological Association, the European Association of Urology and the NCCN endorse RP, LDR and EBRT therapy as appropriate treatment options for IRPC. However, no difference has been observed in OS or CSS among the three approaches in the recent studies [21, 22]. Treatment options for PCa are diverse, and therapeutic decisions are primarily based on the condition of each medical institution and the preferences of doctors/patients [21]. IRPC represents a heterogeneous population for which primary therapy includes AS, RP, EBRT with or without ADT, brachytherapy with or without ADT, or EBRT + BT with or without ADT [23, 24]. AS has been considered as a feasible strategy and is well recommended in low-risk PCa patients with equivalent oncological outcomes [25, 26], nevertheless, the application of AS in IRPC is not yet clear. Currently, NCCN suggests that AS should be considered for IRPC patients who meet the following conditions: (1) the predominant cancer lesion is grade group 1 or 2; (2) the tumor involves less than 50% of the core, (3) the patient only has one NCCN intermediate-risk factor [15]. However, in a recent systematic review and meta-analysis, Baboudjian found that the risk of metastasis and cancer mortality in AS for unselected IRPC patients were significantly higher than those in low-risk PCa patients, highlighting the need to optimize patient selection for patients with intermediate-risk characteristics [27].

Comparing the oncological outcomes of RP and LDR remains a challenge due to differential definitions for recurrence and methodological biases arising from the differences in baseline characteristics, including age, comorbidity and cancer risk features [21, 28–30]. A randomized controlled trial is ideal for comparing treatment modalities [31], nevertheless, studies on comparison of the efficacy between LDR and RP in previously reported literature are retrospective design [21, 32]. Compared with candidates for RP, patients who offered LDR generally tend to be older and have higher comorbidity scores; therefore, a random trial is impractical [29, 30]. Würnschimmel et al. demonstrated in a retrospective single‐center study that low‐ and IRPC patients treated with RP can expect an impressive life expectancy predictions with OS = 90.7% BF ranged from 61.7% to 81.9% in ten-year follow-up. The difference in OS between their study and ours could be explained by the differences in age and race [33, 34]. In addition, previous study did not perform subgroup analysis for low-risk PCa and IRPC patients, whereas our study only focuses on the IRPC. Although RP provides a treatment method for patients with PCa, this surgical approach does not come without significant short- and long-term adverse effects, with decline in sexual function and urinary incontinence being the ones most frequently reported. According to previous studies, the reporting rate of urinary incontinence after RP varies from 5 to 40% [35]. Multiple techniques have been recently applied in order to achieve early recovery of urinary continence after RP, however, a consensus has not yet to be reached regarding which strategy is most effective in facilitating early continence recovery [36]. Erectile dysfunction is another most concerning post-surgical complications after RP, with a prevalence ranging from 10–82% [37, 38]. Oral treatment of phosphodiesterase type 5 inhibitor (PDE5i) is the first line standard care treatment for erectile dysfunction as it is non-invasive and can be provided on demand, nevertheless, PDE5i is ineffective in up to 82% of patients after RP [39]. Thus to guarantee patients’ quality of life without compromising oncology prognosis, it is necessary to explore an optimal substitute for RP [40].

After retrospectively analyzing data from 361 IRPC patients treated in our hospital, we found no statistically significant difference in the cRFS, CSS and OS between the two therapeutic groups. This result was consistent with recent publications in the literature [41–43]. Hamdy et al. also reported 10-year outcomes after monitoring, surgery, or radiotherapy for localized PCa, and highlighted that there was no significant difference in cRFS, CSS and OS between surgery and radiotherapy treatments [22]. However, as his study included patients of all risk categories, we cannot make any conclusions regarding IRPC. Goy et al. statistically analyzed 1503 IRPC patients who underwent treatment from 2004 to 2007 and demonstrated that there was no significant difference between cRFS and CSS, while as for the bRFS, the LDR is significantly higher when compared with other treatment approaches. The 10-year bRFS was 80.2% for LDR, 57.1% for RP and 57.0% for EBRT, *P* = 0.0003 [5]. However, in his study, LDR patients had a significantly smaller proportion than RP (7.3% Vs 54.5%) which might cause bias in the results. In present research, the proportion of patients in both groups was more balanced than that in Goy’s study and our results revealed the 5- and 8-year bRFS rates were 70.2% and 63.1% in the RP group, and 83.2% and 68.9% in the LDR group, respectively. The log-rank test showed that the bRFS for RP was significantly lower than LDR. However, in terms of the pretreatment characteristics, patients treated with LDR were older, experienced longer follow-up times, and had a higher preponderance of combined ADT treatment. Although the clinical stage differed significantly with *P* value of < 0.001 in univariate analysis, our multivariate analysis has proved that the clinical stage is not a variable that could affect the clinical outcome.

Generally, it is hard for studies comparing the prognosis of LDR and RP to unify the ADT treatment plan. To reduce heterogeneity, our study opted to regard ADT application and duration as a risk variable in multivariable analysis and our results indicated that ADT application has limited impact on the prognosis of IRPC according to the results of both multivariate and univariate analysis which met the results of former study [30, 44]. This result can ensure the reliability of our research results to a certain extent. Cindolo et al. indicated that the OS in the super-elderly is also not influenced by persistence and/or adherence [45]. Our findings suggested that LDR may act as a viable alternative to RP for IRPC patients. As neither treatment modality has been proven superior to the other concerning RP and LDR, the optimal treatment for different risk categories in PCa remains debatable. Although our median follow-up time of 54 months was sufficient to identify a significant number of systemic failure events, it may still be too short to achieve mortality results. Therefore, we chose bRFS as the primary evaluation criterion for curative effect. We observed that LDR out performed RP significantly in terms of bRFS, especially, for patients with GS 7, PV > 30 ml, iPSA ≤ 10 ng/ml, clinical T stage with T1c–T2a, any PPBC or unfavorable IRPC. Supported by the results of our study, LDR might be better option than RP in patients with the above conditions. Subgroup analysis revealed that the bRFS of unfavorable IRPC could be significantly improved through LDR compared with RP. All these results could prove that LDR was a considerable option for those with unfavorable IRPC. Similarly, a study conducted by Taussky et al. reported that RP and LDR treatment led to comparable outcomes at 4 years post-treatment in patients with low- and low-intermediate- risk PCa [46]. However, Ferreira et al. found that the 5-year bRFS of patients with early PCa patients who had undergone LDR was significantly higher than those who had received surgery [47]. Furthermore, Ciezki et al. reported that higher bRFS was achieved through LDR and EBRT as compared to RP in the treatment of high-risk PCa [41]. It is worth noting that these studies exhibited significant heterogeneity as different centers employed diverse LDR technologies and comparison methods were varied when comparing the outcomes of RP and LDR [48].

As the previous studies reported, there were many factors affecting the prognosis of PCa, including general situation, tumor stage, tumor `grade, iPSA, age and bone scintigraphy result [49–51]. Ciezki et al. found that clinical stage T3, GS 8 to 10, higher iPSA and more frequent post-treatment PSA testing were all associated with a significantly worse bRFS [41]. Additionally, Taussky et al. reported that younger age, a higher percentage of positive biopsies, and PSA at diagnosis were predictive for BF [46]. On our multivariate analysis, PV ≤ 30 ml, positive margin and PPBC > 50% were associated with significantly worse bRFS. Goy et al. showed that PPBC rate > 50% had a substantial impact on cRFS and CSS [46]. Raison et al. highlighted that positive margin was a strong predictors of biochemical recurrence after RP [52]. While as a reasonable explanation for why PV < 30 ml was associated with worse bRFS still could not be found. A multi-center population based prospective study in the future is warranted to further investigate the relationship between PV and the prognosis of PCa.

Current international and guidelines for post-RP patients with adequate PSA response (< 0.1 ng/mL) or those at high risk of recurrence recommend either adjuvant radiotherapy or early salvage radiotherapy [23]. Nevertheless, it remains controversial whether patients with IRPC should also treated by adjuvant radiotherapy or salvage radiotherapy. Adjuvant radiotherapy is conducted immediately (within 4 –6 months) after RP whereas salvage radiotherapy is given after a period of observation and BF. Adjuvant radiotherapy has been proved to reduce the risk of recurrence after RP. However, three recently published phase III trials (RAVES, RADICALS, GETUG17), indicated clearly that salvage radiotherapy at the time of recurrence may now be regarded as a preferred option in the large majority of patients [53–55]. Therefore, in our present study, adjuvant radiotherapy was applied to guarantee an optimal clinical outcome.

The limitations of this study include the following: (1) baseline characteristics of the two groups did not wholly match. Significant differences were observed in terms of age, TNM staging, ADT duration and adjuvant EBRT application between RP group and LDR group, resulting in a high heterogeneity. Nevertheless, our results to some extent do reflect the actual clinical situation. In clinical practice, older patients tend to prefer LDR, while those with higher TNM staging are more likely to opt for RP. Additionally, patients who receive RP are more inclined to choose adjuvant ERBT as a supplemental therapy. Although the minor misfit is inevitable due to random grouping in a retrospective study, a prospective study comparing eligible patients is still needed to make a more accurate conclusion. As this study aims to provide a guide to aid clinical decision-making at diagnosis, the duration of ADT following initial treatment, which may contribute to survival, was not adjusted. Nevertheless, according to previous studies using models adjusted for risk, ADT was not an independent predictor [30, 44]. (2) The definition of BF is different between RP and LDR group. Although this definition is commonly used in the world [5, 22, 43], there might remain some bias in the interpretation of the results. (3) Fewer deaths in this study means that whether higher bRFS rates observed in patients could translate into superior oncological endpoints is still undetermined; consequently, a more extended observational period is needed for a meaningful comparison of OS. (4) Due to its retrospective nature, co-morbidities of participants in each group were not compared in the present study. In addition, since our study was conducted in a single hospital center, there may be some potential limitations, such as patient selection bias and small sample size. We are currently following up with the included patients to collect relevant clinical data to improve our study in the future.

## Conclusion

In summary, LDR might be a considerable treatment option for IRPC patients, with equivalent rates of cRFS, CSS and OS when compared with RP. Despite the difference in BF definitions, LDR could improve bRFS significantly when compared with RP. PV ≤ 30 ml (*P* < 0.001), positive margin (*P* < 0.001) and PPBC > 50% (*P* < 0.001) were independent predictors for worse bRFS. A longer follow-up may still be required to detect differences in OS between these two treatments.

## Data Availability

The datasets used and/or analyzed during the current study available from the corresponding author on reasonable request.

## Abbreviations

ADT: Androgen deprivation therapy
BF: Biochemical failure
bRFS: Biochemical relapse-free survival
CI: Confidence interval
cRFS: Clinical relapse-free survival
CSS: Cancer-specific survival
EBRT: External beam radiation therapy
GS: Gleason score
HR: Hazard ratio
iPSA: Initial prostate-specific antigen
IRPC: Intermediate-risk prostate cancer
LDR: Low-dose-rate brachytherapy
NCCN: The National Comprehensive Cancer Network
OS: Overall survival
PCa: Prostate cancer
PPBC: Percentage positive biopsy cores
PSA: Prostate-specific antigen
RP: Radical prostatectomy
